# Extracts of *Artocarpus communis* Decrease **α**-Melanocyte Stimulating Hormone-Induced Melanogenesis through Activation of ERK and JNK Signaling Pathways

**DOI:** 10.1155/2014/724314

**Published:** 2014-03-05

**Authors:** Yi-Tzu Fu, Chiang-Wen Lee, Horng-Huey Ko, Feng-Lin Yen

**Affiliations:** ^1^Department of Fragrance and Cosmetic Science, College of Pharmacy, Kaohsiung Medical University, Kaohsiung 80708, Taiwan; ^2^Department of Nursing, Division of Basic Medical Sciences, Research Center for Industry of Human Ecology, Chang Gung University of Science and Technology, Taoyuan 333, Taiwan; ^3^Chronic Diseases and Health Promotion Research Center, Chang Gung University of Science and Technology, Chiayi City 61363, Taiwan

## Abstract

*Artocarpus communis* is an agricultural plant that is also used in folk medicine to prevent skin diseases, including acne and dermatitis. Extracts of *A. communis* have been used to effectively inhibit melanogenesis; however, the antimelanogenesis mechanism of these extracts has not yet been investigated. The present study utilized a cell-free tyrosinase assay as well as **α**-melanocyte stimulating hormone- (-MSH-) induced tyrosinase assay conducted in B16F10 cells, performed a cytotoxicity assay, and determined cellular melanin content to examine the effects of a methanolic extract of *A. communis* (ACM) and various organic partition fractions of *A. communis* on melanogenesis. In addition, we performed western blot analysis to elucidate the mechanism of their antimelanogenesis effect. Our results indicated that, except for the n-hexane extract, ACM and the various partition extracts at noncytotoxic concentrations effectively decreased melanin content and tyrosinase activity by downregulating microphthalmia-associated transcription factor (MITF) and phosphorylated cAMP response element-binding protein (p-CREB). Moreover, ACM and the partition fractions activated phosphorylation of extracellular signal-regulated kinase (ERK) and c-Jun N-terminal kinase (JNK) to inhibit the synthesis of MITF and finally to decrease melanin production. In conclusion, we suggest that noncytotoxic concentrations of ACM and the various partition fractions may be useful as references for developing skin-lighting agents for use in medicines or cosmetics.

## 1. Introduction 

In humans, normal melanin production plays an important photoprotective role against the carcinogenic and deleterious effects of UV radiation and in scavenging toxic drugs and chemicals [1]. However, excessive pigmentation affects the appearance of the skin and can increase the risk of melanoma. Hydroquinone was generally used for treating hyperpigmentation in the past, but it is now banned by the European Committee because of side effects such as perdurable depigmentation and exogenous ochronosis [2]. Kojic acid, another popular skin-lightening treatment that is commonly used to prevent pigmentation, also produces side effects such as dermatitis and erythema due to cytotoxicity [3]. Thus, the development of a safe, effective, and innovative skin-lightening agent with no harmful side effects would be beneficial.

Flavonoids are used in several natural medical therapies for their many beneficial effects, including anticancer and anti-inflammatory actions, as well as for protection against UV radiation [4]. Therefore, flavonoid-rich plants are currently being developed for use as lightening agents. Various species of *Artocarpus *are abundant sources of prenylated flavonoids and derivativesand have therefore been examined phytochemically and biologically for their antifungal [5], antiplatelet [6], anti-inflammatory [7], and radical scavenging activities [8]. *A. communis *(Moraceae), commonly known as the breadfruit tree, is native to Pacific and Tropical Asia, Indonesia, and Papua New Guinea [9]. Breadfruit is a traditional staple food in tropical and subtropical regions and may have medicinal properties. The roots of the breadfruit tree, in folk medicine, are considered useful for treating headaches, beriberi, vomiting, parulis, and edema [10]; whereas the leaves have been traditionally used for alleviating diabetes, hypertension, and cirrhosis of the liver [11], suggesting that *A. communis *is beneficial and pharmacologically active. Thus, the potential for developing *A. communis *as a skin-lightening agent has been examined. However, the biological mechanisms for inhibition of melanogenesis by *A. communis *have not yet been investigated.

The aim of the present study was to elucidate the correlation between cell viability and antimelanogenesis by comparing the total phenolic content, cytotoxicity, and the inhibition of melanogenesis by a methanolic extract of *A. communis *(ACM) and various organic fractions, including n-hexane (ACNH), dichloromethane (ACD), ethyl acetate (ACE), and n-butanol (ACNB). The effects of this extract and these fractions on cell viability were determined using the MTT assay and on tyrosinase activity using a cell-free mushroom tyrosinase assay. We also examined the concordance of safety with the inhibition of melanogenesis by these extracts and fractions by determining the cellular melanin content and the reduction of *α*-melanocyte stimulating hormone- (-MSH-) induced tyrosinase activity in B16F10 cells. In addition, western blot analysis was used to determine tyrosinase expression and tyrosinase-related proteins, including microphthalmia-associated transcription factor (MITF), cAMP response element-binding protein (CREB), tyrosinase-related proteins-1 (TRP-1) and -2 (TRP-2), extracellular signal-regulated kinases (ERK), and c-Jun N-terminal kinase (JNK).

## 2. Materials and Methods

### 2.1. Chemical Reagents

Gallic acid, dimethyl sulfoxide (DMSO), 3-(4,5-dimethylthiazol-2-yl)-2,5-diphenyl tetrazolium bromide (MTT), and phenylmethylsulfonyl fluoride (PMSF) were purchased from Sigma (St. Louis, MO, USA). L-DOPA was purchased from Wako Pure Chemical Industries, Ltd. Trypsin-EDTA was obtained from Biological Industries. Octylphenol ethoxylate (Triton X-100) was purchased from J. T. Baker. All other chemical reagents were of analytical grade and commercially available.

### 2.2. Preparation of *Artocarpus communis* Extracts

The heartwood of *A. communis *was collected from the Tainan District Agricultural Research and Extension Station, COA, in Taiwan in July 2010. Two kg of dry heartwood was immersed in 20 L of methanol for 2 weeks. The extract was filtered through filter paper and then concentrated to dryness under reduced pressure. The dried extract (56.85 g) was dissolved in distilled water (1.5 L) and further partitioned in succession with 1.5 L of n-hexane (for ACNH), dichloromethane (for ACD), ethyl acetate (for ACE), and n-butanol (for ACNB), with each procedure in triplicate. The dry weight of ACNH, ACD, ACE, and ACNB was 2.34 g, 15.63 g, 14.56 g, and 13.35 g, respectively.

### 2.3. Total Phenolic Content Assay

The total phenolic content in ACM and the partition extracts were determined by using the Folin-Ciocalteu reagent according to the method published by Prior et al. (2005) [12]. Gallic acid was used to create a calibration curve for determining the total phenolic content in each sample. All determinations were performed in triplicate.

### 2.4. Mushroom Tyrosinase Assay

The inhibition of mushroom tyrosinase activity was determined using L-DOPA (Wako, Japan) as a substrate. Briefly, 100 *μ*L of 2.5 mM L-DOPA in 67 mM phosphate buffer (pH 6.8), 80 *μ*L of 67 mM phosphate buffer (pH 6.8), and 40 *μ*L of various concentrations (40, 20, 10, and 5 *μ*g/mL) of the different extracts and fractions were added to each well of a 96-well microplate and mixed with gentle shaking at room temperature for 10 min. Then, 40 *μ*L of 100 units/mL of mushroom tyrosinase was added to each well for incubation at room temperature for 60 min. The relative amount of dopachrome formed in the mixture was determined at an absorbance of 475 nm using a microplate spectrophotometer (*μ*Quant, BioTek). Kojic acid at various concentrations (12.5, 25, 50, and 100 *μ*g/mL) was used as a positive control, verifying assay feasibility and performance. The inhibition of tyrosinase activity was calculated as follows:

(1) tyrosinase  activity  (%) =[OD475  of  control−OD475  of  sample]OD475  of  control×100,

where OD475 is the optical density measured at an absorbance of 475 nm.

### 2.5. Cell Culture

B16F10 murine melanoma cells were cultured in Dulbecco's modified eagle medium (DMEM) adjusted to contain 1.5 g/L sodium bicarbonate and supplemented with 10% fetal bovine serum and 1% antibiotic solution containing 100 unit/mL of penicillin G, 100 *μ*g/mL of streptomycin, and 0.025 *μ*g/mL of amphotericin B in an incubator with a humidified atmosphere containing 5% CO_2_ at 37°C.

### 2.6. Cell Viability Assay

Cell viability was determined using the MTT assay to establish the noncytotoxic concentrations of the various *A. communis *extracts in B16F10 cells. The MTT assay is based on the reduction of 3-(4,5-dimethylthiazol-2-yl)-2,5-diphenyl tetrazolium bromide (MTT) to formazan by mitochondrial enzymes in cells. B16F10 cells were seeded in a 96-well microplate (1 × 10^4^ cells/well) and allowed to adhere at 37°C overnight. After incubating the various *A. communis *extracts for 48 h, 100 *μ*L of MTT solution (0.5 mg/mL in DMEM) was added to each well. The cells were incubated at 37°C for an additional 2 h before addition of 100 *μ*L of DMSO to dissolve the formazan precipitates using gentle shaking for 15 min. The absorbance was measured at 575 nm using a microplate spectrophotometer (*μ*Quant, BioTek). Each treatment was repeated three times. Cell viability was calculated with the following formula:

(2) $$\begin{array}{c} \text{cell}\;\text{viability}\left(\%\right)=\frac{\text{OD}575\;\text{of}\;\text{sample}}{\text{OD}575\;\text{of}\;\text{control}}\times 100. \end{array}$$

### 2.7. Melanin Content Assay

B16F10 cells were seeded in 6-well plate (2 × 10^5^ cells/well) and incubated overnight to allow cells to adhere. After treating with various test samples in the presence of 10 nM of *α*-MSH in an incubator for 48 h, cells were washed with PBS and then harvested using trypsin. After centrifugation at 12,000 rpm for 10 min, the supernatant was removed and the cell pellet was lysed with 100 *μ*L of 1 N NaOH. Melanin was dissolved with 10 min of heating at 95°C. The lysate (85 *μ*L) was added to 96-well microplates and the relative melanin content was determined by measuring the absorbance at 415 nm using a microplate spectrophotometer (*μ*Quant, BioTek). The relative melanin content was calculated with the following formula:

(3) $$\begin{array}{c} \text{relative}\;\text{melanin}\;\text{content}\;\left(\%\right)=\frac{\text{OD}415\;\text{of}\;\text{sample}}{\text{OD}415\;\text{of}\;\text{control}}\times 100. \end{array}$$

### 2.8. Cellular Tyrosinase Activity Assay

B16F10 cells were seeded at a density of 2 × 10^5^ cells/well in a 6-well plate that contained 2 mL of DMEM and incubated overnight to allow cells to adhere. After treating with various samples in the presence of 10 nM *α*-MSH and incubating for 48 h, the cells were washed with phosphate buffered saline (PBS) and harvested with trypsin. The solutions were centrifuged at 12,000 rpm for 10 min, and the cell pellets were collected and lysed with lysis buffer (100 *μ*L of 1% Triton X-100 solution and 100 *μ*L of 0.1 mM PMSF). This solution was frozen at −80°C for 30 min and thawed at room temperature for 30 min. This treatment was repeated twice to completely lyse the cells. The lysates were centrifuged at 12,000 rpm for 30 min to obtain the supernatant. The reaction mixture, containing 20 *μ*L of 0.05% L-3,4-dihydroxyphenylalanine (L-DOPA) in 0.1 M sodium phosphate (pH 6.8) and 80 *μ*L of the supernatant (tyrosinase source), was placed in 96-well microplates and incubated at 37°C for 3 h. The optical densities were measured at 492 nm with a microplate spectrophotometer (*μ*Quant, BioTek). The cellular tyrosinase activity was calculated with the following formula:

(4) $$\begin{array}{c} \text{tyrosinase}\;\text{activity}\;\left(\%\right)=\frac{\text{OD}492\;\text{of}\;\text{sample}}{\text{OD}492\;\text{of}\;\text{control}}\times 100. \end{array}$$

### 2.9. Analysis of Protein Expression Using Western Blotting

B16F10 cells were treated with various concentrations of extracts and fractions in the presence or absence of 10 nM *α*-MSH in a 6-well plate for 48 h. The cells were collected and lysed with 200 *μ*L of sample buffer composed of 10% glycerol, 5% *β*-mercaptoethanol, 2% sodium dodecyl sulfate (SDS), and 0.01% bromophenol blue in 62.6 mM Tris-HCl buffer (pH 6.8). The lysates were denatured at 95°C for 10 min and subjected to SDS-polyacrylamide gel electrophoresis (PAGE) using a 12% running gel before being transferred to nitrocellulose membranes. Membranes were incubated for 24 h with the various primary antibodies, including MITF, p-CREB, TYR, TRP-1, TRP-2, p-ERK, p-JNK, and glyceraldehyde 3-phosphate dehydrogenase (GAPDH). The membranes were then incubated with anti-mouse or anti-rabbit horseradish peroxidase antibody for 1 h. The immunoreactive bands of protein were reacted with ECL reagents and photographed using a gel imaging system (Alpha Innotech Co.). All determinations were performed in triplicate.

### 2.10. Statistical Analysis

All data were expressed as the mean ± SD of 3 independent experiments. Comparisons between 2 groups were analyzed using a two-tailed Student's *t*-test with the 2010 version of Microsoft Excel software.

## 3. Results 

### 3.1. Cell Viability of Extracts and Various Fractions from *A. communis* in B16F10 Cells

Cell viability was determined using MTT assay, and noncytotoxic concentrations of the extracts and fractions from* A. communis *were applied in the assays examining melanogenesis. Survival of 90% of the cells (cell viability, CV90) was used to evaluate the safety of ACM and the various fractions. As shown in Table 1, CV90 for ACM, ACNH, ACE, ACNB, and ACD was 23.71, 98.27, 31.97, 29.27, and 8.7 *μ*g/mL, respectively. The fractions were ranked in order of their potency to cause cytotoxicity as ACD > ACM > ACNB > ACE > ACNH. Thus, 20 *μ*g/mL of ACM, 8 *μ*g/mL of ACD, 100 *μ*g/mL of ACNH, and 30 *μ*g/mL of both ACNB and ACE were considered safe and used in the cellular melanin content and tyrosinase activity assays.

### 3.2. Concordance between Inhibition of Tyrosinase Activity in a Cell-Free System and Total Phenolic Content in Various Extracts and Fractions of *A. communis*

**Figure 1:**
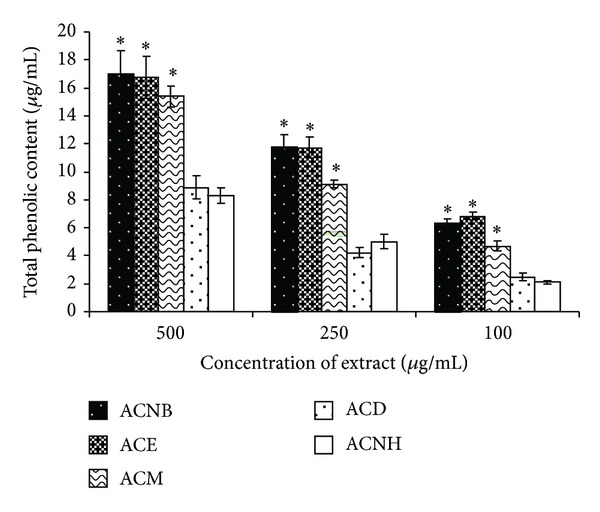
Total phenolic content of *A. Communis *methanol extract and its different partition extracts. Each percentage value was expressed as the means ± SD of three independent experiments. *P < 0.05, significant difference with ACNH.

Previous studies have indicated that the degree of inhibition of melanogenesis by a plant extract, for example, *Magnolia grandiflora,* is directly correlated with its total phenolic content [13]. We found that the total phenolic content of ACM and the partition fractions were ACNB > ACE > ACM > ACD > ACNH (Figure 1). The effects of the extract and various fractions of *A. communis *on the activity of mushroom tyrosinase and on the concentration that inhibited 50% of the mushroom tyrosinase activity (IC50) are shown in Table 2. The results indicated that ACM, ACE, and ACNB inhibited mushroom tyrosinase activity with IC50 values of 27.98, 28.14, and 39.50 *μ*g/mL, respectively, which were more potent than kojic acid (IC50 = 54.72 *μ*g/mL); whereas ACD produced only a mild inhibitory effect (IC50 = 56.87 *μ*g/mL). ACNH did not affect mushroom tyrosinase activity even at a high concentration (400 *μ*g/mL). These results demonstrated that ACM possessed antimelanogenesis activity and that a rank order of efficacy for ACM and the partition fractions to inhibit mushroom tyrosinase activity was ACNB > ACE > ACM > ACD > ACNH.

### 3.3. Cellular Melanin Content and Tyrosinase Activity of the Methanolic Extract and Various Organic Fractions of *A. communis*

**Figure 2:**
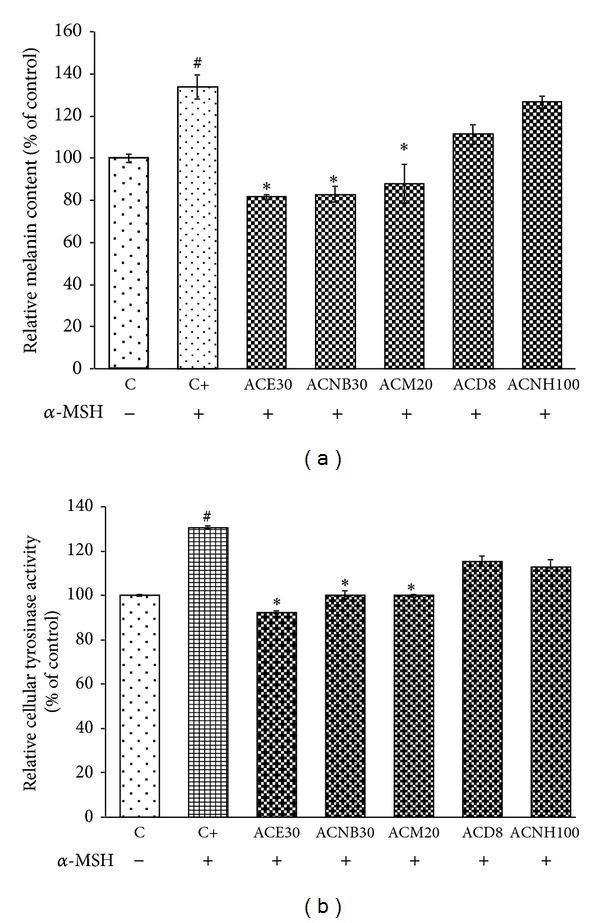
Effects of *A. Communis *extract and its different partition extracts on *α*-MSH-induced cellular melanin content (a) and tyrosinase in B16F10 (b). Each percentage column for treated cells was shown to be relative to that of control. The results have been shown as the means ± SD and are representative of three independent experiments. ^#^ *P* < 0.05 indicates significant difference from control in the absence 10 nM of *α*-MSH; *P < 0.05 indicates significant difference from *α*-MSH treatment control.

The relative cellular melanin content is shown in Figure 2(a).The melanin content was significantly increased (33.61%) by *α*-MSH (10 nM) compared to the control group (*P* < 0.001). ACM (20 *μ*g/mL), ACE (30 *μ*g/mL), ACNB (30 *μ*g/mL), and ACD (8 *μ*g/mL) significantly reduced the melanin content by 45.82, 51.99, 50.78, and 22.25%, respectively (*P* < 0.01). However, ACNH barely inhibited melanin production compared with control in the presence of 10 nM of *α*-MSH. As shown in Figure 2(b), treatment with 10 nM of *α*-MSH significantly increased the activity of tyrosinase compared with that of the control in the absence of *α*-MSH (*P* < 0.05). Treatment with 30 *μ*g/mL of ACE, 30 *μ*g/mL of ACNB, and 20 *μ*g/mL of ACM effectively inhibited *α*-MSH-induced cellular tyrosinase activity by 38.43, 50.78, and 30.48%, respectively. By contrast, ACD and ACNH inhibited tyrosinase activity only slightly better than the control in the presence of 10 nM *α*-MSH.

### 3.4. *A. communis* Extracts Decrease Melanogenesis by Activating MAPK Phosphorylation, Reducing MITF and p-CREB, and Decreasing Tyrosinase Synthesis

**Figure 3:**
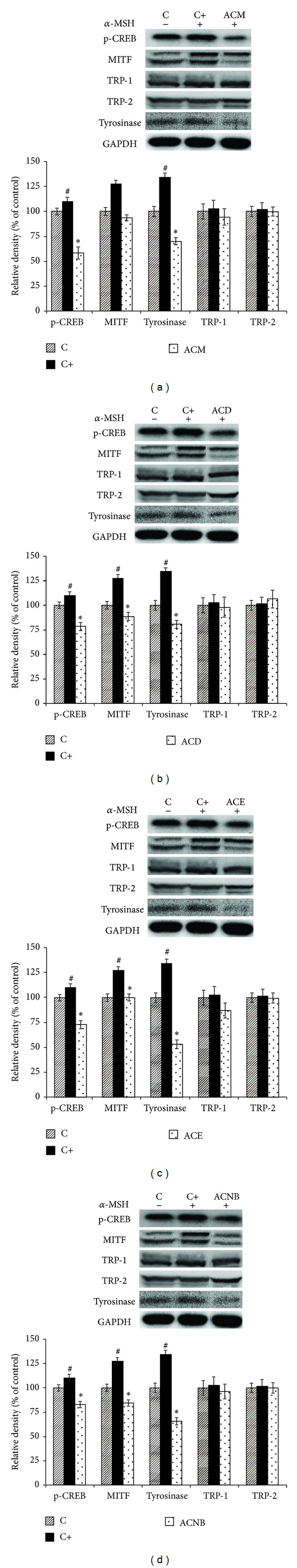
*A. Communis *extract and its different partition extracts downregulate the expression of MITF, p-CREB, and tyrosinase. The B16F10 cells were treated with different extracts (a) ACM, (b) ACD, (c) ACE, and (d) ACNB. ^#^ *P* < 0.05 indicates significant difference from control in the absence 10 nM of *α*-MSH; *P < 0.05 indicates significant difference from *α*-MSH treatment control.

**Figure 4:**
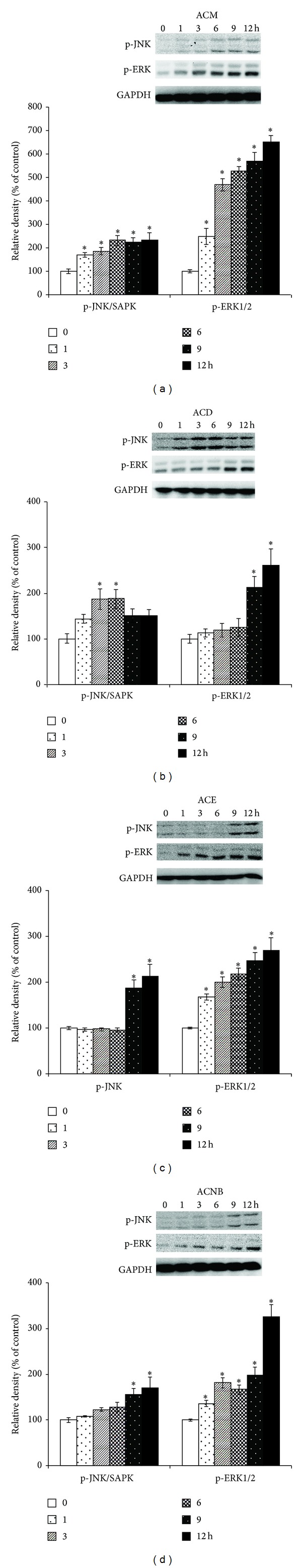
*A. Communis *extract and its different partition extracts downregulate the expression of MITF, p-CREB, and tyrosinase. The B16F10 cells treated with different extracts (a) ACM, (b) ACD, (c) ACE, and (d) ACNB. *P < 0.05 indicates significant difference from 0 h.

Previous studies have demonstrated that a skin-lightening agent can activate ERK and JNK phosphorylation and reduce MITF and p-CREB protein expression to decrease tyrosinase synthesis in *α*-MSH-induced melanogenesis in B16F10 cells [14]. As shown in Figure 3, 20 *μ*g/mL of ACM (Figure 3(a)), 8 *μ*g/mL of ACD (Figure 3(b)), 30 *μ*g/mL of ACE (Figure 3(c)), and 30 *μ*g/mL of ACNB (Figure 3(d)) effectively reduced *α*-MSH-induced MITF protein overexpression, CREB phosphorylation, and tyrosinase protein synthesis (*P* < 0.05), without affecting TRP-1 and TRP-2. In addition, ACM (Figure 4(a)), ACD (Figure 4(b)), ACE (Figure 4(c)), and ACNB (Figure 4(d)) significantly activated the phosphorylation of ERK and JNK in a time-dependent manner to downregulate MITF protein expression (*P* < 0.05).

## 4. Discussion

**Figure 5:**
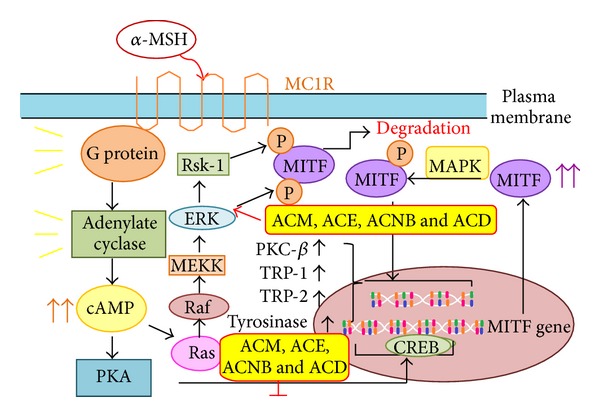
Antimelanogenesis biological mechanism of* A. communis* extracts.

By measuring a decrease in cellular melanin content and tyrosinase activity in B16F10 cells, the present study demonstrated that ACM and the different partition fractions of *A. communis* at concentrations that did not induce cytotoxicity inhibited melanogenesis. This antimelanogenesis activity of ACM and the partition fractions is likely attributable to the total phenolic content. In addition, this study demonstrated that the decrease in tyrosinase synthesis and melanin production by ACM and the various partition fractions is mediated through the activation of ERK and JNK phosphorylation, which downregulates MITF protein expression; see Figure 5.

It is well known that the safety of natural products and pure compounds is a major consideration for using them as drugs, health foods, and cosmetic ingredients. Hydroquinone is a skin-lightening agent for treating melanogenesis-related diseases, such as melasma and postinflammatory pigmentation [15]. However, high concentrations of hydroquinone can induce many side effects due to its cytotoxicity, including skin rash, scaling, and contact dermatitis [16]. Therefore, a good skin-lightening agent should produce no cytotoxicity in vitro. Our study demonstrated that 20 *μ*g/mL of ACM, 8 *μ*g/mL of ACD, 100 *μ*g/mL of ACNH, 30 *μ*g/mL of ACNB, and 30 *μ*g/mL of ACE maintained B16F10 cell viability at 90%. Therefore, B16F10 cells were treated with these noncytotoxic concentrations of extracts and partition fractions, and the melanin content and tyrosinase activity were determined in the cells. We found that noncytotoxic concentrations of ACM, ACE, and ACNB effectively decreased the activity of the rate limiting enzyme, tyrosinase, and reduced the *α*-MSH-induced melanin overproduction in B16F10 cells. Arung et al. have similarly demonstrated that *A. heterophyllus *extract and several flavonoids from the *Artocarpus *plant inhibit melanin biosynthesis in B16 cells [17].

Many studies have indicated that *α*-MSH is a c-AMP activator, stimulating the protein levels of p-CREB and MITF to enhance the synthesis of tyrosinase-related proteins leading to melanogenesis [18]. The present study has also demonstrated that *α*-MSH significantly increased the expression of p-CREB and MITF and enhanced the activity of tyrosinase and the production of melanin. Good skin-lightening agents, such as caffeic acid phenethyl ester [19], *Arthrophytum scoparium *[20], and gingerol [21], have been shown to decrease p-CREB and MITF to prevent melanogenesis. Our results demonstrated that ACM, ACD, ACE, and ACNB at noncytotoxic concentrations can inhibit *α*-MSH-induced p-CREB and MITF to diminish the *α*-MSH-induced melanogenesis in B16F10 cells.

Previous studies have also shown that the MAP kinase family, including ERK and JNK, plays an important role in regulating melanogenesis [22, 23]. Many publications have demonstrated that efficacious inhibitors of melanogenesis activate the phosphorylation of ERK and JNK and result in the phosphorylation of MITF at serine 73, which induces subsequent ubiquitin-dependent proteasomal degradation [24]. In the present study, ACM, ACD, ACE, and ACNB at noncytotoxic concentrations effectively activated the phosphorylation of ERK and JNK in a time-dependent manner. Our results further revealed that ACM, ACD, ACE, and ACNB effectively degraded MITF and decreased the synthesis of tyrosinase and production of melanin through the activation of p-ERK and p-JNK signaling pathways.

In conclusion, the results from the present study suggested that, except for ACNH, ACM and the various partition fractions activated the phosphorylation of ERK and JNK to degrade MITF and decrease the activity of tyrosinase and the production of melanin. Consequently, we suggest that these extracts can be used as skin-lightening agents for treating hyperpigmentation disorder and may be used as an active ingredient in skin care products for preventing the darkening of the skin. However, their safety and efficacy in humans will need to be examined in future studies.

## Figures and Tables

**Table 1:** The cell viability of *Artocarpus communis *extracts.

Sample	CV_90_^a^ (*μ*g/mL)
ACNB	29.27 ± 0.11
ACM	23.71 ± 2.99
ACD	8.7 ± 0.76
ACE	31.97 ± 1.66
ACNH	98.27 ± 2.92

^a^The concentration at cell viability rate of 90%.

**Table 2:** The cell-free tyrosinase inhibitory activity of *Artocarpus communis *methanolic extract and its different fractions.

Sample	IC_50_ (*μ*g/mL)^a^
ACNB	27.98 ± 2.62
ACM	28.14 ± 0.82
ACD	56.87 ± 6.84
ACE	39.50 ± 4.20
ACNH	—^b^
Kojic acid^c^	54.72 ± 5.07

^a^The concentration at inhibitory mushroom tyrosinase activity rate of 50%.

^b^Not detected. There is no inhibitory activity of mushroom tyrosinase at the concentration of 400 *μ*g/mL of ACNH.

^c^Kojic acid as a positive control.
